# Supplementary material for: A computational theory of the subjective experience of flow
Source: Nat Commun. 2022 Apr 26;13:2252. doi: 10.1038/s41467-022-29742-2 (PMC9042870; doi:10.1038/s41467-022-29742-2)
Supplement: Supplementary file 1 — Supplementary Information [file 41467_2022_29742_MOESM1_ESM.docx]

**Supplementary Information for**

A computational theory of the subjective experience of flow

David E. Melnikoff, Ryan W. Carlson, Paul E. Stillman

David E. Melnikoff

Email: [davidemelnikoff@gmail.com](mailto:davidemelnikoff@gmail.com)

**This PDF file includes:**

Supplementary Methods

Supplementary Results

Supplementary Tables 1-4

Supplementary References

**Supplementary Methods**

**Instructions to participants.** Instructions in each experiment were largely identical. Below we include the task instructions for experiment 1 as a representative example (for complete code used to display instructions for each experiment, see <https://github.com/carlsonrw/flowAsMI>):

- *We are designing games that can be used by behavioral scientists to study motivation and decision making. To make the games as engaging as possible, we are getting feedback from people like you.*
- *You will play two different games: the “Green Game [Blue Game]” and the "Blue Game [Green Game]”. Then you will tell us which you found more engaging.*
- *The games are very similar, but their color schemes will help you tell them apart.*
- *Continue to learn about and play the Green Game [Blue Game]. After you finish, you will learn about and play the Blue Game [Green Game].*
- *The goal of the Green Game [Blue Game] is to win as many "10-cent Jackpots" as possible.*
- *For every 10-cent Jackpot you win, you will receive an extra 10 cents. Thus, at the end of the study, you will receive $7 for your participation, plus an additional 10 cents for each 10-cent Jackpot you win.*
- *To win 10-cent Jackpots, you will try to "activate" tiles like this one: [image of grey tile]*
- *The tiles will appear and disappear very quickly. To activate a tile, you must press your SPACE BAR before it disappears. Thus, whenever you see a tile, you should press your SPACE BAR as fast as possible.*
- *In the "Green Game [Blue Game], tiles turn green [blue] if activated. [image of green/blue tile]*
- *If you turn a tile green [blue], your odds of winning a 10-cent Jackpot that trial are [60%, 70%, 80%, 90%, 100%].*
- *If a tile disappears before you turn it green [blue], your odds of winning a 10-cent Jackpot that trial are [40%, 30%, 20%, 10%, 0%].*
- *If you win a 10-cent Jackpot, you'll see this image [image of jackpot]...*
- *...and if you don't win a 10-cent Jackpot, you'll see this image [image of no jackpot]*

*If you have read and understood the instructions, please continue to play the Green Game [Blue Game]. To re-read any of the instructions, press the 'previous' button to navigate back.*

**Supplementary Results**

**Manipulation of** $\boldsymbol{p}_{\boldsymbol{M}}\boldsymbol{(hit)}$ **was successful.** To ensure our that our manipulation of $p_{M}(hit)$ successfully influenced the probability of participants pressing the space bar in time, we compared the target number of hits they were supposed to get to how many times they successfully pressed the space bar in time. Across experiments, participants had the exact target number of hits 42% of the time (experiment 1: 32%, experiment 2: 51%, experiment 3: 53%), were within 3 hits of the target 85% of the time (experiment 1: 76%, experiment 2: 93%, experiment 3: 93%), and within 5 hits of the target 90% of the time (experiment 1: 84%, experiment 2: 95%, experiment 3: 97%). Overall, then, it appears that our manipulation of $p_{M}(hit)$ was successful.

**Response time analyses for experiment 1.** Experiment 1 was programmed such that response time was only recorded for a hit; if the space bar was pressed before the grey square disappeared RT was recorded correctly, but if participants pressed the space bar after the grey square disappeared (as was often the case on when the square was only presented for 250 ms), RT was not recorded. This introduced the possibility of bias in our results, as the RT data is based on a subset of trials, and as such we do not report these results in the main text. Nevertheless, $I(M;E)$ had a significant, negative effect on RT in experiment 1 (*b* = -.05, *SE* = .01, *t*(471.96) = -4.98, *p* < .001), replicating our findings from experiments 2 and 4. The effect of $I(M;E)$ on RTSD was negative but not significant (*b* = -.06, *SE* = .04, *t*(584.14) = -1.54, *p* = .124). As in the main text, these analyses included $p_{M}(hit)$ as a covariate.

**Attention effects do not depend on the inclusion of** $\boldsymbol{p}_{\boldsymbol{M}}\left( \boldsymbol{hit} \right)$ **as a covariate.** As explained in the main text, we included $p_{M}\left( hit \right)$ as a covariate in our analyses of attention. To further confirm that our results hold when this covariate is removed, we reran all analyses in which $I(M;E)$ was a significant predictor of RT or RTSD, this time without$p_{M}\left( hit \right)$ as a covariate. $I(M;E)$ remained a significant predictor of RT (experiment 1: *b* = -.04, *SE* = .01, *t*(520.95) = 3.8, *p* < .001; experiment 2: *b* = -.06, *SE* = .01, *t*(284.04) = -5.69, *p* < .001; play condition of experiment 4: *b* = -.07, *SE* = .01, *t*(536.01) = 5.97, *p* < .001) and RTSD (experiment 2: *b* = -.24, *SE* = .05, *t*(339.94) = 4.77, *p* < .001; play condition of experiment 4: b = -.13, SE = .04, t(635.41) = 3.71, p < .001).

**Adjusting for skill-challenge balance.** When flow was simultaneously regressed on $I\left( M;E \right)$ and skill-challenge balance, all effects of $I\left( M;E \right)$ remained significant (experiment 1: *b* = .77, *SE* = .13, *t*(404.77) = 5.82, *p* < .001; experiment 2: *b* = .74, *SE* = .18, *t*(291.86) = 4.18, *p* < .001). These analyses also revealed significant, positive effects of skill-challenge balance (experiment 1: *b* = .28, *SE* = .04, *t*(479.9) = 6.27, *p* < .001; experiment 2: *b* = .25, *SE* = .06, *t*(354.16) = 3.92, *p* < .001).

We also explored how adjusting for skill-challenge balance influenced the effects of $I\left( M;E \right)$ on attention. First, we simultaneously regressed RT on $I\left( M;E \right)$ and skill-challenge balance ($p_{M}\left( hit \right)$ was not included as a covariate). All effects of $I\left( M;E \right)$ remained significant such that higher $I\left( M;E \right)$ corresponded to faster responding (experiment 1: *b* = -.04, *SE* = .01, *t*(517.44) = -3.81, *p* < .001; experiment 2: *b* = -.06, *SE* = .01, *t*(281.98) = -5.27, *p* < .001). Greater skill-challenge balance, on the other hand, was associated with significantly slower responding in experiment 2 (*b* = .01, *SE* = .004, *t*(340.21) = 3.57, *p* < .001), and had no effect on RT in experiment 1 (*b* = .004, *SE* = .003, *t*(674.14) = 1.14, *p* = .254). The significant effect of skill-challenge balance on RT in experiment 2 was eliminated after adjusting for $p_{M}\left( hit \right)$ (see Fig. 5A, main text).

Next, we repeated the above analyses, but with RTSD as the outcome. After adjusting for skill-challenge balance, the significant effect of $I\left( M;E \right)$ on RTSD in experiment 2 remained significant (*b* = -.23, *SE* = .05, *t*(339.15) = -4.49, *p* < .001), and the non-significant effect of $I\left( M;E \right)$ on RTSD in experiment 1 remained non-significant (*b* = -.06, *SE* = .04, *t*(583.4) = -1.56, *p* = .120). In these analyses, skill-challenge balance had no effect on RTSD in experiment 1 (*b* = -.01, *SE* = .01, *t*(713.17) = -.97, *p* = .335), and had a significant, positive effect on RTSD in experiment 2 (*b* = .03, *SE* = .02, *t*(431.79) = 1.99, *p* = .048). The significant effect of skill-challenge balance on RTSD in experiment 2 was eliminated after adjusting for $p_{M}\left( hit \right)$ (see Fig. 5A, main text). Together, these results suggest that increasing $I\left( M;E \right)$ boosts attention independent of skill-challenge balance.

Lastly, we explored how adjusting for skill-challenge balance influenced the effects of $I\left( M;E \right)$ on enjoyment in experiment 2. First, we simultaneously regressed continuous enjoyment ratings on $I\left( M;E \right)$ and skill-challenge balance ($p_{M}\left( hit \right)$ was not included as a covariate). In the main text, we reported that the effect of $I\left( M;E \right)$ on enjoyment was not significant, but after adjusting for skill-challenge balance, it became significant (*b* = .46, *SE* = .17, *t*(299.81) = 2.78, *p* = .006) such that as $I\left( M;E \right)$ increased, so did enjoyment. This analysis also revealed a significant, positive effect of skill-challenge balance (*b* = .47, *SE* = .06, *t*(369.93) = 7.86, *p* < .001). Next, we simultaneously regressed choice (i.e., which game participants would prefer to play again) on two variables, one denoting to the degree to which $I\left( M;E \right)$ differed across the two games, and one denoting the degree to which skill-challenge balance differed across the two games. A greater difference in both $I\left( M;E \right)$ (*b* = 1.08, *SE* = .32, *Z* = 3.37, *p* < .001) and skill-challenge balance (*b* = .87, *SE* = .14, *Z* = 6.24, *p* < .001) corresponded to a greater likelihood of choosing the game that dominated in that category.

Overall, these results suggest that $I\left( M;E \right)$ influences self-reported flow, attention, and task enjoyment over and above the impact of skill-challenge balance.

**Adjusting for expected value.** All effects of $I\left( M;E \right)$ on flow remained significant when flow was simultaneously regressed on $I\left( M;E \right)$ and expected value (experiment 1: *b* = .75, *SE* = .14, *t*(408.57) = 5.42, *p* < .001; experiment 2: *b* = .66, *SE* = .18, *t*(293.92) = 3.67, *p* < .001; play condition of experiment 4: *b* = .46, *SE* = .12, *t*(525.06) = 3.97, *p* < .001). The independent effect of expected value on flow was not significant in experiment 1 (*b* = .58, *SE* = .33, *t*(417.77) = 1.73, *p* = .084) or experiment 2 (*b* = .47, *SE* = .44, *t*(306.07) = 1.06, *p* = .289), but it did have a significant, positive effect on flow in the play condition of experiment 4 (*b* = 2.05, *SE* = .31, *t*(565.59) = 6.5, *p* < .001).

We also explored how adjusting for expected value and skill-challenge balance influence the effects of $I\left( M;E \right)$ on attention. First, we simultaneously regressed RT on $I\left( M;E \right)$ and expected value ($p_{M}\left( hit \right)$ was not included as a covariate). All effects of $I\left( M;E \right)$ remained significant such that higher $I\left( M;E \right)$ corresponded to faster responding (experiment 1: *b* = -.05, *SE* = .01, *t*(479.1) = -4.9, *p* < .001; experiment 2: *b* = -.06, *SE* = .01, *t*(272.71) = -5.99, *p* < .001; play condition of experiment 4: *b* = -.06, *SE* = .01, *t*(525.01) = 5.91, *p* < .001). Greater expected value, on the other hand, was associated with significantly *slower* RTs (experiment 1: *b* = .21, *SE* = .02, *t*(500.1) = 8.56, *p* < .001; experiment 2: *b* = .19, *SE* = .03, *t*(281.99) = 7.72, *p* < .001; play condition of experiment 4: *b* = .06, *SE* = .03, *t*(569.31) = 2.07, *p* < .039). A likely reason for this reversal is that expected value is highly positively correlated $p_{M}\left( hit \right)$ at *r* = .90 (unlike $I\left( M;E \right)$, which was uncorrelated with $p_{M}\left( hit \right)$), so as expected value increased, the need to respond quickly decreased. Indeed, the effect of expected value on RT is eliminated after adjusting for $p_{M}\left( hit \right)$ (see Fig. 5A, main text).

Next, we repeated the above analyses, but with RTSD as the outcome. After adjusting for expected value, $I\left( M;E \right)$ remained a non-significant predictor of RTSD in experiment 1 (*b* = -.06, *SE* = .04, *t*(585.2) = -1.46, *p* = .145), and a significant, negative predictor of RTSD in experiment 2 (*b* = -.24, *SE* = .05, *t*(336.42) = -4.78, *p* < .001) and the play condition of experiment 4 (*b* = -.13, *SE* = .04, *t*(636.54) = 3.68, *p* < .001). In contrast, expected value had a non-significant effect on RTSD in experiment 1 (*b* = -.15, *SE* = .10, *t*(614.12) = -1.49, *p* = .138), a significant, positive effect on RTSD in experiment 2 (*b* = .3, *SE* = .12, *t*(354.3) = 2.46, *p* = .015), and a non-significant effect on RTSD in experiment 4 (*b* = -.12, *SE* = .1, *t*(723.22) = 1.24, *p* = .217). In experiment 2, the positive effect of expected value on RTSD was eliminated after adjusting for $p_{M}\left( hit \right)$ (see Fig. 5A, main text). Together, these results suggest that while increased $I\left( M;E \right)$ leads to greater attention on the task at hand, the same is not true of expected value.

Lastly, we explored how adjusting for expected value influenced the effects of $I\left( M;E \right)$ on enjoyment in experiment 2 and the play condition of experiment 4. First, we simultaneously regressed continuous enjoyment ratings on $I\left( M;E \right)$ and expected value ($p_{M}\left( hit \right)$ was not included as a covariate). In the play condition of experiment 4, the independent effect of $I\left( M;E \right)$ on enjoyment was positive and significant (b = .35, SE = .12, t(518.43) = 2.92, p = .004), and in experiment 2, it was positive but not significant (*b* = .31, *SE* = .17, *t*(301.53) = 1.81, *p* = .072). In both experiments, the independent effect of expected value was significant such that as expected value increased, so did enjoyment (experiment 2: *b* = 1.9, *SE* = .43, *t*(315.1) = 4.46, *p* < .001; play condition of experiment 4: b = 3.94, SE = .33, t(556.1) = 12.12, p < .001). Next, in experiment 2, we regressed choice (i.e., which game participants would prefer to play again) on two variables, one denoting to the degree to which $I\left( M;E \right)$ differed across the two games, and one denoting the degree to which skill-challenge balance differed across the two games. A greater difference in both $I\left( M;E \right)$ (*b* = .95, *SE* = .33, *Z* = 2.91, *p* = .004) and expected value (*b* = 6.7, *SE* = .94, *Z* = 7.09, *p* < .001) corresponded to a greater likelihood of choosing the game that dominated in that category.

Overall, these results suggest that $I\left( M;E \right)$ influences self-reported flow, attention, and task enjoyment over and above the impact of expected value.

**Exploratory analyses.** In addition to the measures reported in the main text, we collected three other measures in an exploratory fashion. Specifically, in experiment 2, we measured (*i*) need for cognition, (*ii*) construal level, and (*iii*) time perception.

***Need for Cognition****.* We explored the possibility that $I\left( M;E \right)$ predicts enjoyment only among people who are high in “need for cognition,” the dispositional tendency to engage in and enjoy thinking [1]. To test this possibility, we included the 18-item short Need for Cognition scale [1]. For this measure, participants read and rated how well a series of 18 statements described their personality. For instance, participant read statements such as “*I like complex problems more than simple problems*.” and “*Thinking is not my idea of fun*.”, and responded on a 5-point scale ranging from 1 (“*Extremely Uncharacteristic of Me*”) to 5 (“*Extremely Characteristic of Me*”). Need for cognition did not moderate the effect of $I\left( M;E \right)$ on enjoyment (continuous measure: *b* = .61, *SE* = .34, *t*(312.29) = 1.81, *p* = .071; binary choice: *b* = -.5, *SE* = .53, *z* = .96, *p* = .337).

***Construal Level****.* We explored the possibility that $I\left( M;E \right)$ influences the level of abstractness or concreteness at which people construe their tasks [2]. To test this possibility, we included questions in experiment 2 that probed how abstractly or concretely each tile game was construed. Specifically, after both games, we asked participants to indicate “*Which of the following better describes the activity of playing the Blue Game [Green Game]*?” for the following two construal judgments: (*i*) a 7-point scale ranging from the concrete construal “*Activating Tiles*” to the abstract construal “*Winning Jackpots*”; and (*ii*) another 7-point scale ranging from the concrete construal “*Pressing My SPACEBAR*” to the abstract construal “*Earning Money*”. We regressed the mean of these two items on$I\left( M;E \right)$ and found no effect of $I\left( M;E \right)$ on construal level (*b* = -.19, *SE* = .25, *t*(378.91) = .73, *p* = .468).

***Time Perception****.* We explored the possibility that $I\left( M;E \right)$ influences the subjective experience of time. Specifically, after each tile game in experiment 2, we asked participants “*How long did it feel like the Blue Game [Green Game] lasted?*”. Participants used a continuous slider to indicate how long each game seemed to last in minutes from 0 minutes to 8 minutes (in 10 second increments).$I\left( M;E \right)$ had no effect on time estimates (*b* = -1.1, *SE* = .87, *t*(305.48) = 1.27, *p* = .206).

**Deviations from pre-registration.** In our pre-registration plans for experiments 2 and 3, we indicated that we would regress our key dependent measures on $p_{E|M}(jackpot|hit)$ and the quadratic of $p_{M}(hit)$. A linear combination of these terms is roughly equal to $I\left( M;E \right)$, so we reasoned that both terms should be significant if an outcome is a function of $I\left( M;E \right)$ *per se* rather than its constituent variables. The same reasoning led us to indicate that we would regress $I\left( M;E \right)$ on controllability (as a proxy to $p_{E|M}(jackpot|hit)$; the greater the value of $p_{E|M}(jackpot|hit)$, the more controllable the tile game should feel) and the quadratic of skill-challenge balance (as a proxy to $p_{M}(hit)$; the further the value of $p_{M}(hit)$ from .5, the more the tile game should feel too easy or too hard). Instead of running these analyses, we ran the “entropy-based analyses” reported in the main text, which is the superior strategy due to the fact that $I\left( M;E \right)$ is *exactly* equal to $H\left( M \right)-H(M|E)$, and only *approximately* equal to a linear combination of $p_{E|M}(jackpot|hit)$/controllability and the quadratic of $p_{M}(hit)$/skill-challenge balance.

Also, we had to make several judgement calls regarding exclusions and model specification. Our ultimate choices, which are reported in the main text, at times deviated from our preregistrations. To ensure that our results are robust to different choices in data preprocessing and model specification, we conducted a multiverse analysis of experiment 1 (which was not preregistered) and experiments 2 and 3 (which included deviations from preregistration) [3].

**Multiverse analysis.** A multiverse analysis involves running a statistical test across a “multiverse” of possible data sets, each of which is obtained from a different set of decisions about preprocessing steps and model specification. The robustness of a result is quantified in terms of the frequency with which it appears across the multiverse; the greater the proportion of data sets in which a result is observed, the more robust the result.

Each decision point we used to construct our multiverse is given in Supplementary Table 2. Results of the multiverse analysis, broken down by dependent variable, are given in Supplementary Table 3 (experiment 1 and 2) and Supplementary Table 4 (experiment 3). Overall, our analyses appear highly robust to experimenter decisions. In experiments 1-2, self-reported flow was a significant, positive effect of $I\left( M;E \right)$ in 254 out of 256 models (99.2%, with the other 2 models marginally significant). In experiment 3, all 64 models found that the mixture game (where $I\left( M;E \right)$ was highest) had significantly greater flow then the punishment and neutral games, the in none of the models did flow differ, either significantly or marginally, across the punishment and neutral games (which had the same amount of $I\left( M;E \right)$). In experiments 1-2, $I\left( M;E \right)$ significantly predicted reduced RT in all 512 models. RTSD was slightly less robust, with $I\left( M;E \right)$ significantly predicting reduced RTSD in 287 of 512 models (56.1%, with 372 – 72.7% – of models marginally significant). In experiment 2, 62.5% of the models (10 of 16) found a significant relationship between the difference in $I\left( M;E \right)$ in the two games and participants’ subsequent choice about which to play again, and 87.5% (14 of 16) found a marginal relationship. The least robust association was between enjoyment and$I\left( M;E \right)$: in experiment 2, $I\left( M;E \right)$ significantly corresponded with enjoyment in only 1 of the 64 models (1.56%, 12 of 64 – 18.8% – were marginally significant). In experiment 3, while participants enjoyed the mixture game significantly more than the punishment game in all 32 models, participants enjoyed the mixture game significantly more than the neutral game in 6 (18.8%) models (10 models, 31.2%, marginally significant). In no model was enjoyment greater in the neutral game versus the mixture game, either marginally or significantly. Together, these findings suggest that our results with self-reported flow and attention (as indexed by RT and RTSD) are robust, whereas the link between $I\left( M;E \right)$ and enjoyment is less so, possibly due to enjoyment being relatively more influenced by monetary outcomes on the task.

**Supplementary Table 1.** Principal Components Analysis for experiments 1 and 2

|  | **Experiment 1** | | **Experiment 2** | |
| --- | --- | --- | --- | --- |
| **Item** | **Factor 1** | **Factor 2** | **Factor 1** | **Factor 2** |
| Engaging | .78 | .49 | .85 | .38 |
| Engrossing | .88 | .34 | .89 | .33 |
| Immersive | .83 | .37 | .79 | .47 |
| Addictive | .41 | .91 | .38 | .92 |

*Note.* Values denote factor loadings for each flow item.

**Supplementary Table 2.** Modeling criteria for multiverse analysis

| **Exclusion Criteria** | **Composite Variables** | **Control Variables** |
| --- | --- | --- |
| Exclude those with mean RT outside the IQR range | Self-reported Flow: use the scale with the “addictive” item removed | Controlling for game number |
| Exclude those who had more than 5 ‘hits’ fewer or greater than their manipulation called for | RT and RTSD: remove trials that were less than 100ms or those which followed a ‘too fast’ trial | Controlling for the linear effect of p(m) (Experiments 1-2 only) |
| Exclude those who failed to fill out all DVs | RT and RTSD: log transform |  |
| Exclude those who were too fast on 10 or more trials |  |  |

*Note.* RT analyses only conducted for Experiments 1-2.

**Supplementary Table 3.** Multiverse results for experiments 1 and 2

| **Dependent Variable** | **% of analyses with two-tailed *p* < .05 (*p* < .1)** | **# of**  **models** |
| --- | --- | --- |
| Flow | 99.2% (100%) | 256 |
| RT | 100% (100%) | 512 |
| RTSD | 56.1% (72.7%) | 512 |
| Enjoyment | 1.56% (18.8%) | 64 |
| Choice | 62.5% (87.5%) | 16 |

*Note*. Percentage of models in the multiverse for which $I\left( M;E \right)$ had a significant (two-tailed *p* < .05) or marginal (two-tailed *p* < .1) effect on each dependent variable in the anticipated direction: positive for flow, enjoyment, and choice, and negative for RT and RTSD.

**Supplementary Table 4.** Multiverse results for experiment 3

| **Dependent Variable** | **Contrast** | **% of analyses with two-tailed *p* < .05 (*p* < .1)** | | **# of**  **models** |
| --- | --- | --- | --- | --- |
| Flow | Mixture > Punishment | 100% (100%) | | 64 |
|  | Punishment >  Mixture | 0% (0%) | | 64 |
|  | Mixture >  Neutral | 100% (100%) | | 64 |
|  | Neutral >  Mixture | 0% (0%) | | 64 |
|  | Neutral >  Punishment | 0% (0%) | | 64 |
|  | Punishment >  Neutral | 0% (0%) | | 64 |
| Enjoyment | Mixture > Punishment | 100% (100%) | | 32 |
|  | Punishment >  Mixture | 0% (0%) | | 32 |
|  | Mixture >  Neutral | 18.8% (31.2%) |  | 32 |
|  | Neutral >  Mixture | 0% (0%) |  | 32 |
|  | Neutral >  Punishment | 21.9% (40.6%) |  | 32 |
|  | Punishment >  Neutral | 0% (0%) |  | 32 |

*Note*. Percentage of models in the multiverse for which each contrast was significant (two-tailed *p* < .05) or marginal (two-tailed *p* < .1) for each dependent variable. For example, the first row indicates the percentage of models in which flow was significantly (and marginally) greater in the mixture game versus the punishment game, and the second row indicates the percentage of models in which flow was significantly (and marginally) greater in the punishment game versus the mixture game.

**Supplementary References**

1. Cacioppo, J. T., Petty, R. E., & Feng Kao, C. (1984). The efficient assessment of need for cognition. *Journal of personality assessment*, *48*(3), 306-307.
2. Trope, Y., & Liberman, N. (2010). Construal-level theory of psychological distance. *Psychological review*, *117*(2), 440-463.
3. Steegen, S., Tuerlinckx, F., Gelman, A., & Vanpaemel, W. (2016). Increasing transparency through a multiverse analysis. *Perspectives on Psychological Science*, *11*(5), 702-712.
