# Supplementary material for: A computational theory of the subjective experience of flow
Source: Nat Commun. 2022 Apr 26;13:2252. doi: 10.1038/s41467-022-29742-2 (PMC9042870; doi:10.1038/s41467-022-29742-2)
Supplement: Supplementary file 3 — Reporting Summary [file 41467_2022_29742_MOESM3_ESM.pdf]

nature portfolio

Corresponding author(s): David E. Melnikoff

Last updated by author(s): Feb 1, 2022

Reporting Summary

Nature Portfolio wishes to improve the reproducibility of the work that we publish. This form provides structure for consistency and transparency in reporting. For further information on Nature Portfolio policies, see our Editorial Policies and the Editorial Policy Checklist.

Statistics

For all statistical analyses, confirm that the following items are present in the figure legend, table legend, main text, or Methods section.

n/a	Confirmed	
☐	☒	The exact sample size (*n*) for each experimental group/condition, given as a discrete number and unit of measurement
☐	☒	A statement on whether measurements were taken from distinct samples or whether the same sample was measured repeatedly
☐	☒	The statistical test(s) used AND whether they are one- or two-sided<br>*Only common tests should be described solely by name; describe more complex techniques in the Methods section.*
☐	☒	A description of all covariates tested
☐	☒	A description of any assumptions or corrections, such as tests of normality and adjustment for multiple comparisons
☐	☒	A full description of the statistical parameters including central tendency (e.g. means) or other basic estimates (e.g. regression coefficient) AND variation (e.g. standard deviation) or associated estimates of uncertainty (e.g. confidence intervals)
☐	☒	For null hypothesis testing, the test statistic (e.g. *F*, *t*, *r*) with confidence intervals, effect sizes, degrees of freedom and *P* value noted<br>*Give P values as exact values whenever suitable.*
☒	☐	For Bayesian analysis, information on the choice of priors and Markov chain Monte Carlo settings
☐	☒	For hierarchical and complex designs, identification of the appropriate level for tests and full reporting of outcomes
☐	☒	Estimates of effect sizes (e.g. Cohen's *d*, Pearson's *r*), indicating how they were calculated

Our web collection on statistics for biologists contains articles on many of the points above.

Software and code

Policy information about availability of computer code

Data collection	All data were collected using jsPsych 6.1.0. Custom code is available at https://github.com/carlsonrw/flowAsMI.
Data analysis	All data were analyzed using R (version 3.6) in RStudio (version 1.3). Custom code is available at https://osf.io/23vst/.

For manuscripts utilizing custom algorithms or software that are central to the research but not yet described in published literature, software must be made available to editors and reviewers. We strongly encourage code deposition in a community repository (e.g. GitHub). See the Nature Portfolio guidelines for submitting code & software for further information.

Data

Policy information about availability of data

All manuscripts must include a data availability statement. This statement should provide the following information, where applicable:

- Accession codes, unique identifiers, or web links for publicly available datasets
- A description of any restrictions on data availability
- For clinical datasets or third party data, please ensure that the statement adheres to our policy

All data described in the current manuscript are available in the OSF repository, https://osf.io/23vst/.

Field-specific reporting

Please select the one below that is the best fit for your research. If you are not sure, read the appropriate sections before making your selection.

☐ Life sciences ☒ Behavioural & social sciences ☐ Ecological, evolutionary & environmental sciences

For a reference copy of the document with all sections, see nature.com/documents/nr-reporting-summary-flat.pdf

Behavioural & social sciences study design

All studies must disclose on these points even when the disclosure is negative.

Study description	All studies are quantitative experimental; quantitative outcomes were collected from subjects randomly assigned to experimental conditions.
Research sample	All samples are convenience samples of participants located in the United States recruited online through Prolific.co. A slight majority of participants identified as female and most were between the ages of 20 and 40 (experiment 1: N = 365, 62% Female, mean age = 35; experiment 2: N = 249, 68% Female, mean age = 26; experiment 3: N = 236, 52% Female, mean age = 32; experiment 4: N = 941, 59% female; mean age = 40; experiment 5: N = 397, 60% female; mean age = 37). Our sampling strategy was chosen to promote statistical power and generalizability by enabling the recruitment of large numbers of demographically diverse participants.
Sampling strategy	Participants were randomly assigned to experimental conditions. Power analysis was used to determine the minimum sample size necessary to have an 80% chance of detecting a small-to-medium sized effect at $p < .05$ (two-sided).
Data collection	All experiments were created using jsPsych 6.1.0, and were administered online through Prolific.co. Experimenters were blind to experimental condition, and all participants completed the experiments without an experimenter present.
Timing	Experiment 1: 3/2/2020 to 3/4/2020; Experiment 2: 5/8/2020 to 5/11/2020; Experiment 3: 6/25/2020*; Experiment 4: 5/16/2021*; Experiment 5: 6/27/2021*. Dates marked with * indicate that all data was collected within a single day.
Data exclusions	Data were excluded from analysis if the respondent failed one or more attention checks. Exclusions were implemented to minimize the likelihood of Type 1 and Type 2 error due to random or inattentive responding. Exclusions were planned in advance, and preregistered in experiments 2-5. Overall, 9.7% of respondents (N = 209) were excluded.
Non-participation	Non-participation data is not available because no data were recorded from participants who exited an experiment prior to completion.
Randomization	Subjects were randomly assigned to groups using a random number generator.

Reporting for specific materials, systems and methods

We require information from authors about some types of materials, experimental systems and methods used in many studies. Here, indicate whether each material, system or method listed is relevant to your study. If you are not sure if a list item applies to your research, read the appropriate section before selecting a response.

Materials & experimental systems

n/a	Involved in the study	
☒	☐	Antibodies
☒	☐	Eukaryotic cell lines
☒	☐	Palaeontology and archaeology
☒	☐	Animals and other organisms
☐	☒	Human research participants
☒	☐	Clinical data
☒	☐	Dual use research of concern

Methods

n/a	Involved in the study	
☒	☐	ChIP-seq
☒	☐	Flow cytometry
☒	☐	MRI-based neuroimaging

Human research participants

Policy information about studies involving human research participants

Population characteristics	See above
Recruitment	All participants were recruited online through Prolific.co. This approach is associated with relatively low risk of systematic bias due to self-selection.
Ethics oversight	Yale University's Institutional Review Board

Note that full information on the approval of the study protocol must also be provided in the manuscript.
